# A Review of Therapeutic Aptamer Conjugates with Emphasis on New Approaches

**DOI:** 10.3390/ph6030340

**Published:** 2013-03-19

**Authors:** John G. Bruno

**Affiliations:** Operational Technologies Corporation, 4100 NW Loop 410, Suite 230, San Antonio, TX 78229, USA; E-Mail: brunobiotech@gmail.com; Tel.: +1-210-731-0015; Fax: +1-210-731-0041.

**Keywords:** antibiotic-resistant, aptamer, cancer, complement, DNA, nanoparticle, opsonization, PCR, SELEX, siRNA

## Abstract

The potential to emulate or enhance antibodies with nucleic acid aptamers while lowering costs has prompted development of new aptamer-protein, siRNA, drug, and nanoparticle conjugates. Specific focal points of this review discuss DNA aptamers covalently bound at their 3' ends to various proteins for enhanced stability and greater pharmacokinetic lifetimes *in vivo*. The proteins can include Fc tails of IgG for opsonization, and the first component of complement (C1q) to trigger complement-mediated lysis of antibiotic-resistant Gram negative bacteria, cancer cells and possibly some parasites during vulnerable stages. In addition, the 3' protein adduct may be a biotoxin, enzyme, or may simply be human serum albumin (HSA) or a drug known to bind HSA, thereby retarding kidney and other organ clearance and inhibiting serum exonucleases. In this review, the author summarizes existing therapeutic aptamer conjugate categories and describes his patented concept for PCR-based amplification of double-stranded aptamers followed by covalent attachment of proteins or other agents to the chemically vulnerable overhanging 3' adenine added by Taq polymerase. PCR amplification of aptamers could dramatically lower the current $2,000/gram cost of parallel chemical oligonucleotide synthesis, thereby enabling mass production of aptamer-3'-protein or drug conjugates to better compete against expensive humanized monoclonal antibodies.

## 1. Introduction

Polyclonal antibodies or antisera have long been used as simple passive neutralizing agents for toxins or venoms. More recently humanized monoclonal antibodies have become popular for their many benefits as bio-therapeutic agents or specific “magic bullets.” Unfortunately, xenogeneic and allogeneic antisera can lead to serum sickness or anaphylactic shock, if used *in vivo* more than once [1] and humanized monoclonal antibodies are very expensive due to their long and arduous development cycles [2,3,4]. In addition, even fully “humanized” monoclonal antibodies can be immunogenic especially in their complementarity determining regions (CDRs) or hypervariable antigen binding sites [5].

Antibodies and their more recent molecular “cousins” the nucleic acid-based aptamers generally cannot kill target cells by themselves. Antibodies and aptamers merely bind to target cells or molecules against which they are developed with high affinity and specificity and either gain entry to the cell via receptor-mediated endocytosis [6] or peptide-mediated cell entry mechanisms [7,8,9] or mark the target cell for surface attack. It is, of course, the Fc tail of an antibody or a conjugated toxic molecule on an antibody or aptamer which brings about target cell destruction in the form of enhanced phagocytosis or opsonization [10], complement-mediated lysis [11,12,13,14,15], inhibition of protein synthesis [16], or other lethal mechanisms. These facts enable a bio-molecular engineer to couple antibodies or aptamers to a variety of toxic molecules or other effectors such as drugs [17,18,19], radioisotopes [20,21], phototoxic dyes and quantum dots [22,23,24,25,26,27,28,29] and various other nanoparticles [30,31,32,33,34,35,36,37] or small interfering RNA (siRNA) molecules [38,39,40,41,42] to achieve target cell destruction via the conjugate alone or in conjunction with physical forces including light and microwaves. This article summarizes many of the well-known methods for producing cytotoxic aptamer conjugates, but also focuses on lesser known DNA aptamer-3'-protein [13,43,44] and drug (e.g., ibuprofen, naproxen, *etc.*) conjugates in order for the aptamer-drug conjugates to bind and “hitch a ride” on protective albumins and other proteins in serum [45,46,47,48,49,50].

**Figure 1:**
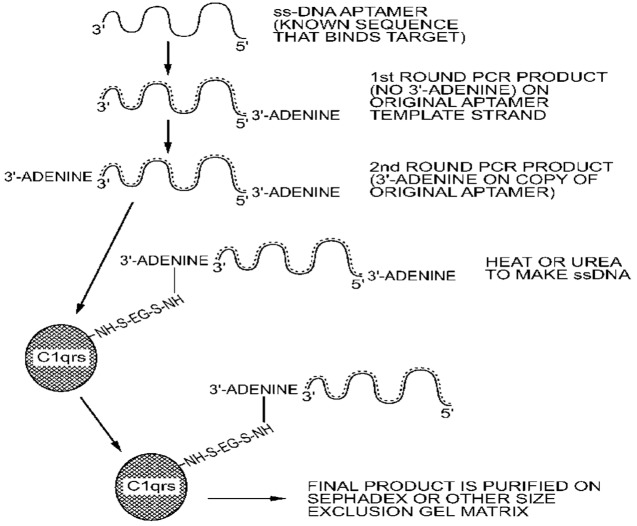
The author's patented [13] chemical scheme for attachment of proteins such as Fc fragments for opsonization [10] and C1qrs to invoke complement-mediated lysis of Gram negative bacteria [11], cancer cells [12,15], or parasites by direct coupling to the unpaired 3' adenine overhang on double-stranded (ds) PCR products imposed by Taq DNA polymerase. Although a sulfo-EGS bifunctional linker (ethylene glycol bis(sulfosuccinimidylsuccinate)) is shown, low levels of glutaraldehyde [43] or other bifunctional linkers could be used as well.

The advent of humanized monoclonal antibodies (hu mAbs) such as Herceptin^®^ for breast cancer treatment has validated the efficacy of immunotherapy [14,51,52]. Unfortunately, the retail cost of humanized monoclonals to patients is nearly prohibitive [2,3,4] and their development is extremely long and arduous, which may account for the high cost. Various aptamer conjugates hold the promise of being relatively inexpensive, if produced by the Polymerase Chain Reaction (PCR; Figure 1), and highly selective “magic bullet” alternatives for immunotherapy. Aptamer-3'-Fc or C1q conjugates in particular could be developed rapidly against tumor cells, parasites and viruses, because some of these agents mutate over time and the innate immune system may need to be redirected during the critical antibody development period prior to seroconversion (*i.e.*, act as passive immunity “bridges to life” [10,11,53,54,55]). Unfortunately, while very attractive in theory, the continual rapid development of new aptamer sequences to bind changing epitopes on mutating target cells seems unlikely to receive FDA approval, except perhaps for terminal patients. Of course, antibody technology is faced with the same “moving target” mutational drift dilemma. Fortunately, this is not an immensely significant problem and most of the well-known cancer markers and pathogen surface antigens are fairly stable.

The potential importance of aptamer conjugates as therapeutic agents is underscored by a number of efforts in recent years, including the formation of a company called Altermune, LLC by Nobel Prize winner Kary Mullis who has patented [56] and demonstrated the use of aptamer-alpha gal conjugates to redirect the immune system to prevent inhalation anthrax [57] and potentially other diseases. In addition, numerous groups have demonstrated the prevention or retardation of bacterial and viral diseases or biotoxin effects using aptamers for passive immunity *in vitro* and *in vivo* [10,11,53,54,55,58,59]. Even NASA has at least postulated the use of aptamer technology on board future spacecraft to counteract the effects of lethal extraterrestrial “Andromeda strain” microbes or latent viruses in astronauts which may exert their pathogenic effects after astronauts are stressed in the microgravity environment of deep space for a prolonged period of time [60].

## 2. Strategies for Conferring Greater Stability and Pharmacokinetic Lifetimes to Aptamers

The largest historical obstacle to the widespread use of aptamers and their predecessors (antisense oligonucleotides) as therapeutic agents has been their stability *in vivo*. The problem is really two-fold in nature: (1) DNA and RNA are susceptible to exonucleases and endonucleases in blood and other body fluids, and (2) because of their small size (generally about 25–30 kD), aptamers are rapidly cleared from circulation by the kidneys and other major organs, thereby drastically reducing their pharmacokinetic lifetime and therapeutic potential [61].

Several different biochemical approaches have been taken to enhancing the lifetime of aptamers *in vivo*. One approach has been to modify the phosphate backbone with sulfur atoms in place of some oxygen atoms to produce phosphorothioates or the so called “thio aptamers” [62] Another strategy has been to modify key sites on the sugar moieties with 2'-fluoro or 2'-O-methyl groups to confer resistance to nucleases [63]. Likewise, blockage of the 3' or 5' ends of aptamers with small molecules such as biotin or the use of inverted bases on the ends has been semi-successful at retarding the enzymatic degradation due to serum nucleases and these modifications are used in the one FDA-approved anti-VEGF aptamer called Macugen^®^. The limitations to these modifications are that the modified sugars, especially if internal to the aptamer chain and associated with a binding site, may affect binding affinity or specificity toward the target. An interesting alternative to these chemical modifications is the RNA “Spiegelmer” (German for “mirror oligomer”) which utilizes the L-enantiomer of ribose to inhibit nuclease digestion [64,65]. While aptamer-peptide conjugates have the potential to facilitate aptamer entry into target cells [7,8,9], such conjugates do not add much weight. In fact, none of the chemical modifications or alternative strategies for enhancement of aptamer stability described thus far addresses the need for larger size or greater mass to avoid rapid renal or other organ clearance.

**Figure 2:**
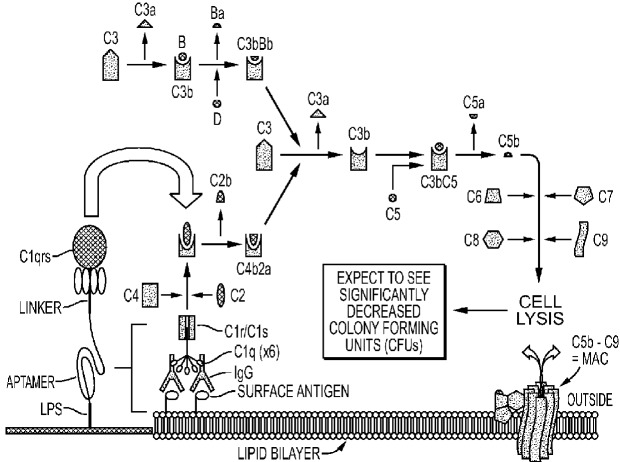
Schematic of the putative DNA aptamer-C1qrs conjugate-mediated triggering of the classical complement system to kill Gram negative bacteria and other thin-walled (cancer and some parasite) target cells by complement-mediated lysis.

**Figure 3:**
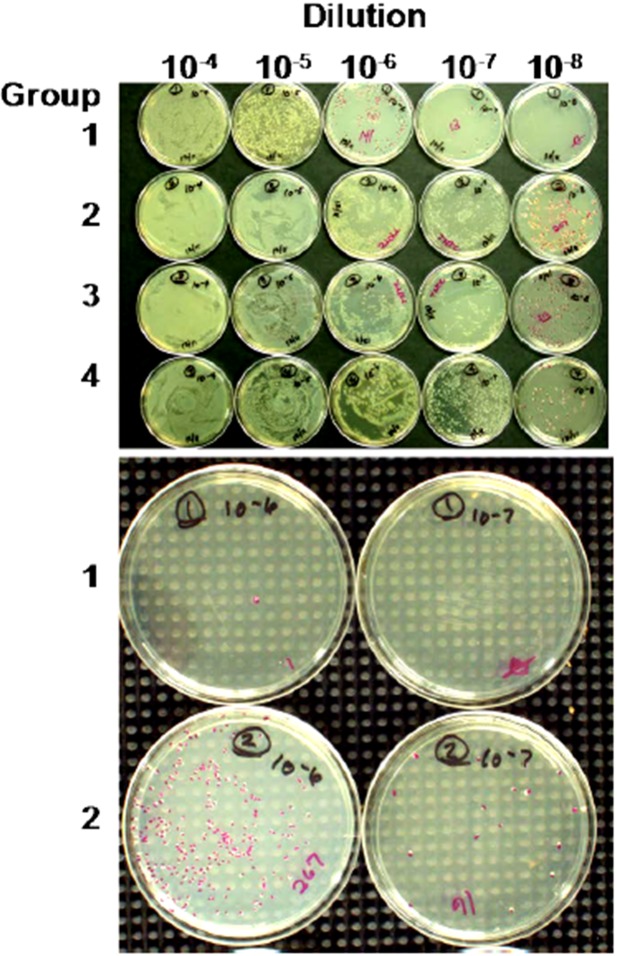
Spread plates from an anti-*E. coli* O111 lipopolysaccharide (LPS) aptamer-C1q killing experiment [11]. The “antibiotic” effect due to aptamer-C1q triggering of the complement system is especially visible in the lower panel where the full test Group 1 shows few, if any, colonies while control Group 2 shows a noteworthy number of colonies across the same two dilutions. The compositions of each group were as follows: Group 1-full test group with aptamer-C1q conjugate and all other complement components. Group 2-control group for the alternative pathway which contained no aptamer-C1q and simply assessed bacterial colony counts in the presence of human serum. Group 3- was a positive growth control group with no serum or aptamer-C1q added. Group 4- contained bacteria treated only with the aptamer-C1q conjugate, but may have triggered some bacterial killing due to contaminating traces of other complement or serum proteins. Ten-fold serial dilutions are shown across the top of the top panel. The bottom panel shows only the 10^−6^ and 10^−7^ dilutions for Groups 1 and 2 from a second experiment.

**Figure 4:**
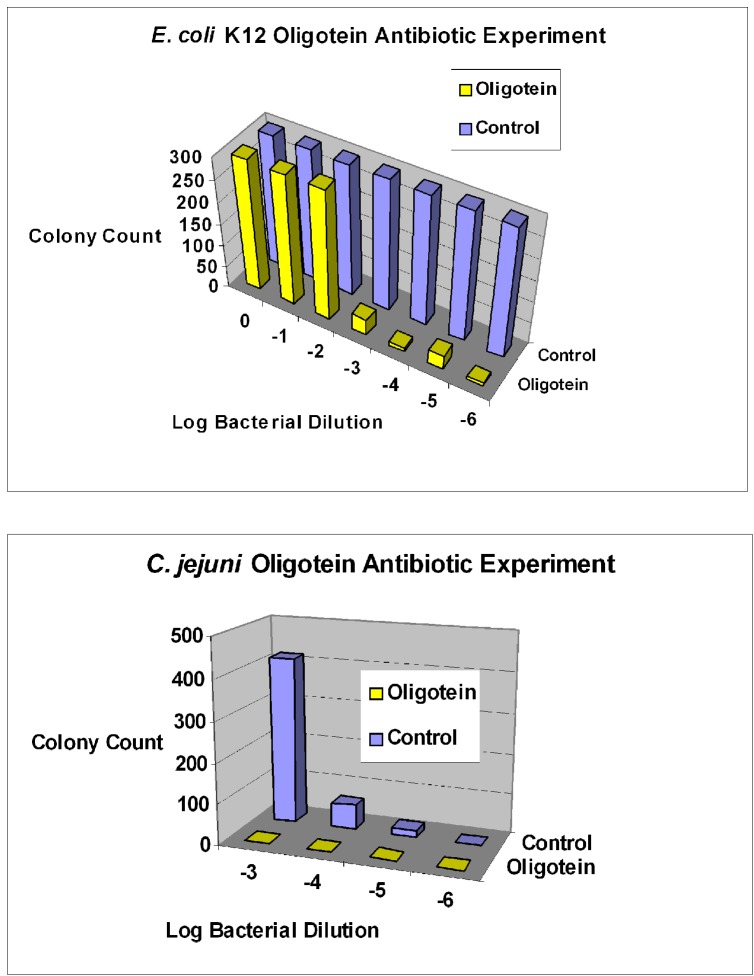
Further examples of aptamer-biotin-streptavidin-C1q killing of Gram negative bacterial species. Top panel shows results for *E. coli* strain K12 with clear cytopathic effect at 10^−3^ dilution and beyond. Bottom panel shows killing of *Campylobacter jejuni* at the same dilutions. “Oligoteins” indicate where aptamer-biotin-streptavidin-C1q conjugates were used.

To address the size issue, some groups have added innocuous polyethylene glycol (PEG) to one end of an aptamer [66,67,68]. Dougan *et al.* [69] added streptavidin to the 3'-biotinylated ends of aptamers to block the main exonuclease in serum (Exonuclease I), thus extending the lifetimes of aptamers *in vivo* while adding significant mass to slow renal clearance. The author's group has added functional proteins to its aptamer-protein chimeras (dubbed “oligoteins,” Figure 1) such as the Fc tail of IgG for opsonization [10] or C1q for induction of complement-mediated lysis [11,12,13,15,44] of thin-walled target cells (Figure 2). Since the membrane attack complex (MAC) which results from complement activation and inserts deadly pores in target cells is only about 15 nm deep, it cannot kill Gram positive bacteria which can possess cell walls up to 80 nm thick, but the MAC can kill Gram negative antibiotic-resistant bacteria (a major cause of sepsis-related deaths; Figure 3 and Figure 4) [44]. Aptamer induction of MAC pores could also kill cancer cells [12,15] and some types of parasites during susceptible phases of their life cycles [70] and when they emerge from their host cells.

The major barrier to killing cancer cells and some parasites by the aptamer-Fc or aptamer–C1q conjugate induction of the complement system is the innate ability of some cells to defeat complement activation. Cancer cells, in particular, possess membrane complement regulatory proteins (mCRPs such as CD46, CD55 and CD59) on their surfaces to ultimately thwart MAC pore formation and only allow for about 50%–60% cancer cell kill rates *in vitro* [12,14,15,44,70]. Perhaps the best way to counteract the effects of mCRPs will be to develop new aptamers against them to block their activity and enable higher kill rates in combination with aptamer-Fc or aptamer-C1q conjugates targeted to cancer-specific surface markers.

While some parasites such as *Leishmania* can be highly susceptible to complement-mediated lysis during certain stages of their life cycles (e.g., the promastigote stage) immediately after infection of the host’s blood, these same parasites can coat themselves in C3 protein from the complement cascade and erythrocytes to protect themselves and gain entry to phagocytes where they thrive [71]. Many parasites also take on thicker and more resilient cell walls or plasma membranes in oocyst or cyst-like stages or are protected for long periods inside host cells. Hence, while there are brief windows of opportunity to kill parasites with aptamers by coupling to complement-mediated lysis, those windows are small and can be subverted by the parasites. Therefore, it seems more likely that aptamer-drug or other toxic payload conjugate endocytosis strategies would have a greater chance of success against most parasites.

## 3. General and Novel Attachment Chemistries

Nucleic acid-drug, protein or other molecular conjugation chemistry may seem trivial, but it often is not simple on the industrial scale. Numerous groups have developed and optimized small-scale conjugation approaches involving 3' or 5' primary amines coupled to various other molecules during chemical synthesis of nucleic acids or by classic carbodiimide [72], aldehyde [13,43,44], diazonium, or other approaches which take advantage of the much greater chemical reactivity of primary alkyl amine tags *versus* aryl amines on the nucleotides themselves.

While conjugation may be fairly straightforward on a small analytical scale or during chemical DNA synthesis, the scale-up to industrial mass production can be difficult, expensive and even prohibitive. Aptamers are generally inexpensive on the milligram synthesis scale ( ~50 cents per base). However, because chemical synthesis of DNA is governed by a 99% coupling efficiency for the growing oligonucleotide chain with yields dropping off greatly at greater than 70 bases (*i.e.*, (0.99)^7^ = 0.495 or 49.5% yield), the commercial synthesis of lengthier ( >70 base) SELEX DNA templates was previously discouraged by oligonucleotide synthesis vendors. While shorter aptamers (60–70 bases) may be acceptable for small molecule (drug, dye, vitamin, *etc.*) targets, longer “linked” aptamers appear to be preferable, because they have demonstrated greater affinity, avidity and specificity for more complex antigens composed of several epitopes [73,74,75,76]. This is not surprising when one considers that nature has evolved 110 amino acid complementarity determining regions (CDRs) in antibody chains having three hypervariable binding sites linked together to form a multivalent binding molecule. Lengthier aptamers (up to 200 bases) are now being routinely synthesized on the mg scale. But, no aptamers can be mass produced at the gram-scale without expensive parallel chemical synthesis leading to a cost of ~$2,000 per gram [63].

This fact prompted the author's group to investigate a concept pioneered by Vandalia Research, Inc. (Vandalia, WV, USA) to mass produce aptamers and other DNA molecules cheaply by polymerase chain reaction (PCR) using their large-capacity Triathalon^®^ PCR system [77]. The enzymatic synthesis of DNA aptamers by use of PCR can clearly reduce the price of mass produced aptamers for the pharmaceutical industry, but the products are double-stranded (ds) and single-stranded (ss) aptamers are typically the active forms. The ds to ss conversion can be accomplished by heating or chemical means to break hydrogen bonds between the strands followed by affinity-based purification of the correct, functional, or “sense” strand. The author's group used the fact that double-stranded PCR products contain a chemically vulnerable unpaired overhanging adenine (A) on the 3' end to attach to proteins by various means including low levels of glutaraldehyde at cold temperatures [43,44]. This patented approach [13] could someday expand the use of lengthier *in vivo* stabilized aptamer-3'-protein conjugates by enabling their industrial scale mass production at reasonable costs. 

## 4. Aptamer-Nanoparticle Conjugates

### 4.1. Aptamer-Magnetic Nanoparticle (MNP) Conjugates

Aptamers themselves are typically quite good at homing to their intended targets even in complex solutions including the *in vivo* milieu. But, magnetic nanoparticles can be used as the primary method of physically directing aptamer-MNPs and drug payloads to their target cells or tissues as well by use of an external magnetic field (“magnetic focusing”) which “drags” the MNPs and their complexes to any desired and accessible location in the body [78] where aptamer-MNPs could act as “nanosurgeons” [79]. Superparamagnetic Iron Oxide Nanoparticles (SPIONs) consist of α-Fe_2_O_3_ (hematite), γ- Fe_2_O_3_ (maghemite) and Fe_3_O_4_ (magnetite) of 1-200 nm or greater diameters [78]. SPIONs of 10-100 nm have the greatest lifetimes *in vivo* because they are large enough to avoid renal clearance, but small enough to evade phagocytosis [78]. SPIONs currently have a large diversity of surface coatings [78] for chemical functionalization and aptamer attachment, if desired. MNPs can be used for magnetic hyperthermia (MHT) in conjunction with external alternating magnetic fields (AMFs) of 88.9 kHz to 13.56 MHz [80,81,82]. By comparison, most microwave ovens utilize radiofrequency radiation of 915 MHz or 2,450 MHz. The concept is to heat targeted tumors to between 41 °C and 43 °C because some types of tumor cells are more sensitive to such elevated temperatures than surrounding tissues which do not suffer much collateral damage [83,84]. These temperatures bring about a number of changes detrimental to the cell cycle and can induce apoptosis [85]. Other types of NPs including gold NPs and quantum dots (QDs) have also been used in conjunction with radiofrequency (RF) radiation to kill tumors *in vivo* at least in animal models [86,87,88].

### 4.2. Aptamer-Quantum Dot (QD) and Photosensitizer Conjugates

As aforementioned, Quantum dots (QDs) and other nanoparticles can be used in RF fields to kill target cells. However, QDs themselves are composed of toxic metals such as cadmium and selenium and are therefore cytotoxic to a wide variety of prokaryotic and eukaryotic cells [22,23,24,25,89,90,91] even without an electromagnetic field. QDs appear to be even more toxic when brought in intimate contact with bacteria after conjugation to antibodies [23] (and presumably aptamers) or internalized by other target cells [90,91]. QDs are thought to be strong free radical (reactive oxygen species ROS; *e.g*., singlet oxygen) generators [22,26,27,28] especially when coupled with photosensitizer ligand dyes such as Rose Bengal or chlorin e6 [92,93]. Regardless of the exact mechanisms, QDs are clearly potential cytotoxic components to pair with aptamers in highly lethal conjugates and QDs have the added advantage of enabling deep tissue imaging to determine if the conjugate has reached its target *in vivo* [94].

Ferreira, *et al.* [24] have demonstrated a greater than 500-fold increase in killing of cancerous epithelial cells with a mucin-specific aptamer labeled with chlorin e(6) at its 5' end when excited by a helium cadmium laser. The work of Ferreira, *et al.* [24] and others has thus made the QD component unnecessary and paved the way for a simple class of aptamer-dye conjugates useful against a variety of superficial cancers that are accessible to light even deep in the body (e.g., mucosa of the gastrointestinal tract) when fiber optics are used.

### 4.3. Aptamer-Carbon Nanotube (CNT) Conjugates

A class of nanoparticles that clearly deserves consideration for incorporation into aptamer conjugates is that of carbon nanotubes (CNTs). Liu *et al.* [35] have reported the remarkable ability of single-walled carbon nanotubes (SWCNTs) to lance and kill bacteria in a concentration-dependent manner by themselves when shaken to cause collisions between the bacteria and SWCNTs. It therefore follows that aptamer attachment to SWCNTs might better direct the impaling action of SWCNTs. One can even envision “nanobots” (microscopic “robots”) capable of specific aptamer-based attachment to target cells followed by ATP hydrolysis-driven insertion of CNTs via “nano-motors” to kill target cells by osmotic damage much as the immune system directs complement-mediated lysis via MAC pores [13]. Additionally, the hollow lumens of CNTs can be loaded with drug or toxin molecules for slow timed release of drugs [95] or perhaps even osmotically pumped into target cells when backed by a hypotonic fluid environment or some other thermodynamically favorable pumping mechanism.

### 4.4. Aptamer-Gold Nanoparticle Conjugates

Although used more for detection and diagnosis, several groups have reported various therapeutic schemes for using aptamer-gold and silver nanorods or gold nano “popcorn” having “hot spots” at the ends of the popcorn spikes for targeted photothermal killing of cancer cells [30,32,33,96,97]. These approaches have two major advantages over aptamer-QD conjugate systems. First, gold nanoparticles are generally not very toxic *in vivo*, at least not when compared to QDs. Second, 3' or 5' thiol-functionalized aptamers are readily available to enable facile spontaneous conjugation to gold nanoparticles with size-exclusion chromatography or other simple purification methods. The natural affinity of sulfur for gold makes chemical attachment methods quite facile when compared to the more complex attachment chemistries used for nucleic acids and functionalized QD shells. A third, lesser known, advantage to using aptamer-coated gold or other nanoparticles which Professor Tan's group at the University of Florida has astutely highlighted in the literature is that of increased collective affinity (~26-fold increased affinity when a nanoparticle is used *versus* the individual aptamer's measured affinity) [32]. The classic immunologist may refer to this increased collective affinity as “avidity.” Regardless of the nomenclature, the effect of enhanced affinity is a very good aspect for future therapeutic potential of aptamer-nanoparticle conjugates. 

### 4.5 Aptamer-Chitosan Nanoparticle Conjugates

Chitosan and its family of derivatives (chitosans) originate from chitin, the very tough polymer of *N*-acteyl-d-glucosamine which confers great strength to insect and shellfish exoskeletons. The highly cationic nature of chitosans can disrupt cell membranes making chitosan nanoparticles cytotoxic [98]. This same highly positive charge density also makes chitosans very potentially useful for drug and aptamer delivery [99,100]. Indeed, chitosans and chitosan nanoparticles have long been investigated for their potential to cross the blood-brain barrier, if properly targeted using magnetic nanoparticles [101], antibodies, or possibly aptamers [102]. 

## 5. Aptamer-Drug and Toxin Conjugates

Numerous groups have demonstrated that certain receptor-binding aptamers can greatly aid internalization of drugs such as doxorubicin into cancer cells via endocytosis [6,16,17,18,19]. Chu *et al.* demonstrated several years ago that they could effectively kill prostate cancer cells *in vitro* using aptamer-carbodiimide-gelonin conjugates to inhibit protein synthesis in target cells [16]. Hence, aptamer-drug conjugates are currently the subjects of intense study.

An interesting novel approach which is just beginning to emerge is the conjugation of simple drugs such as ibuprofen and naproxen known to bind serum albumins in the blood to aptamers to enable the aptamers to “hitch” an exonuclease-protected “ride” on albumin with the added benefit of increased mass for the overall aptamer-drug-albumin conglomerate [45,46,47,48,49]. The postulated aptamer-drug conjugate may also be able to offload from the albumin carrier at sites of inflammation. The same effects could be accomplished with aptamer-cholesterol conjugates and cholesterol binding proteins in the blood. Although, frequent and high dose administration of any conjugates containing cholesterol seems ill-advised for other health reasons.

## 6. Other Aptamer Conjugates

There are numerous other approaches to aptamer conjugation which may be of therapeutic value. This “miscellaneous” category includes aptamer-radioisotope conjugates [20,21], aptamer-drug-loaded liposomes [103] or high-capacity drug-carrying dendritic structures [104,105], and the aforementioned aptamer-siRNA conjugates [38,39,40,41,42]. The aptamer-siRNA category is particularly interesting from the standpoint that either an RNA aptamer can be extended to include the small interfering RNA or the siRNA can hybridize to one end of a DNA aptamer for potential future release inside a target cell [38,42]. Such “conjugates” or chimeras can be easily and naturally formed by transcription or simple hybridization. One problem with the aptamer-siRNA conjugate approach has been molecular engineering of each component to avoid inhibition or loss of either the aptamer’s or siRNA’s functionality due to hybridization between the components. Very recently Berezhnoy *et al.* [38] have reported that fusion of a siRNA to the 3' end of a DNA aptamer minimized or eliminated such negative effects on each component of the conjugate.

## 7. Conclusions and Future Directions

In this final section, some interesting emerging directions for aptamer and aptamer conjugate development and improvement are discussed. While this review touched on aptamer-siRNA conjugates, it has yet to mention the most obvious aptamer conjugate embodiment which is that of an aptamer being conjugated to another aptamer, wherein one aptamer may be used for targeting and the other to bring about some therapeutic effect. The so called bivalent or multivalent aptamer is beginning to emerge in the literature and receive attention for its greater affinity [73,74,75,76] and potentially greater specificity against complex target “antigens” [74,106,107]. The argument for binding to complex antigens having several distinct epitopes is entirely logical in that a linked aptamer can bind various epitopes and thereby achieve synergistic increases in affinity and avidity or overall binding strength. In addition, when multiplied together, multivalent aptamer specificity for various binding sites leads to a much lower probability of binding the wrong or unintended target. By comparison to forensic or paternity DNA fingerprinting analysis, the probability of matching multiple alleles is multiplicative and leads to virtual certainty of matches. Similarly, the specificity of longer linked or multivalent aptamers [73,74,75,76,106,107] is sure to increase with every successful new aptamer binding site which is proximal to the previously bound epitope of a complex antigen. Of course, naturally selected lengthier (100–200 base) aptamers which could cover several epitopes at once on a complex antigen would be preferable to relying on luck to pick the correct shorter aptamers or aptamer binding sites to link together for optimal binding of complex antigens post-selection. Nature appears to have evolved the same strategy as multivalent aptamers in the form of antibody complementarity determining regions (CDRs) which are 110 amino acids in length and contain several hypervariable binding sites for complex antigens. Perhaps, future aptamer researchers and developers should take a lesson from nature and emulate the length of the CDR or surpass it, which is now quite possible with most oligonucleotide vendors which no longer limit oligonucleotide length to 70 bases.

Consideration of the plentiful amino acid monomer selection (20 amino acids diversity) *versus* the paucity of natural nucleotides (four nucleotides for DNA or RNA) as building blocks of antibody and aptamer polymers respectively brings to mind another future area for aptamer and aptamer conjugate improvement, namely introduction of exotic amino-, carboxyl-, thio- or other modified bases to expand the repertoire of binding possibilities. The incorporation of exotic or modified nucleotides has been accomplished for catalytic DNAzymes and some aptamers [108,109] whether by chemical synthesis or the use of substrate-permissive DNA polymerases lacking editing functions (e.g., Deep Vent exo-). However, incorporation of modified bases is almost certainly a dual-edged sword since DNA or RNA polymers containing exotic bases will undoubtedly be recognized as foreign or non-self entities by the immune system, thereby making normally non-immunogenic aptamers, much more immunogenic *in vivo*. Still, the prospect for improved binding or even cytotoxic activity from the aptamer itself, should drive this area of research.

One other aspect for potential future consideration especially for longer multivalent aptamers might be internal stabilization of aptamer secondary and tertiary structures. Aptamers should always be selected at or near the temperature of their intended use. Thus, any aptamer intended for therapeutic use should be affinity selected at 37 °C to maximize correct 3-dimensional conformations for target binding. However, even if selected at the optimal working temperature, aptamers may “collapse” to a lower energy conformational state during cold storage and not be able to regain their optimal folded conformation unless reheated and allowed to cool again. To avoid such potential thermally-induced conformational problems, future aptamer research and development of longer aptamers should focus on understanding aptamer 3-dimensional structures and then possibly stabilizing them with specific hydrogen or disulfide bonds as occurs in nature to stabilize protein tertiary and quaternary structures.

Although lacking a crystal ball, the author believes it is not difficult to predict that aptamer-drug, siRNA, and Fc conjugates are the most likely candidates to advance towards eventual FDA approval for treatment of a variety of maladies. This prediction is based on the relative ease of conjugate production with good quality control and obvious similarity of aptamer-Fc and some other conjugates to actual antibodies. FDA-approved pharmaceuticals must of course be efficacious and safe. While many of the more exotic aptamer-nanoparticle or QD conjugates described herein are highly effective and may have applications in more radical therapies, they may not be as safe as aptamer conjugates which more closely emulate natural antibodies and may therefore these more contrived and exotic conjugates may not gain FDA approval. The recent demise of Archemix Corporation, although quite disheartening to members of the nucleic acid therapeutics community, should not dampen enthusiasm for aptamers and their conjugates. At present, numerous aptamer clinical trials are in progress around the world [110,111]. Almost certainly a few of these aptamer trials will be successful and ultimately someday result in better health care.

## References

[1] Hansel T.T., Kropshofer H., Singer T., Mitchell J.A., George A.J. (2010). The safety and side effects of monoclonal antibodies. Nat. Rev. Drug Discov..

[2] Hedden L., O’Reilly S., Lohrisch C., Chia S., Speers C., Kovacic L., Taylor S., Peacock S. (2012). Assessing the real-world cost-effectiveness of adjuvant trastuzumab in HER-2/neu positive breast cancer. Oncologist.

[3] Jeyakumar A., Younis T. (2012). Trastuzumab for HER2-positive metastatic breast cancer: clinical and economic considerations. Clin. Med. Insights Oncol..

[4] Perez-Ellis C., Goncalves A., Jacquemier J., Marty M., Girre V., Roché H., Brain E., Moatti J.P., Viens P., le Corroller-Soriano A.G. (2009). Cost-effectiveness analysis of trastuzumab (Herceptin) in HER2-overexpressed metastatic breast cancer. Am. J. Clin. Oncol..

[5] Harding F.A., Stickler M.M., Razo J., DuBridge R.B. (2010). The immunogenicity of humanized and fully human antibodies: Residual immunogenicity resides in the CDR regions. MAbs.

[6] Meng L., Yang L., Zhao X., Zhang L., Zhu L., Zhu H., Liu C., Tan W. (2012). Targeted delivery of chemotherapy agents using a liver cancer-specific aptamer. PLoS One.

[7] Bolhassani A. (2011). Potential efficacy of cell-penetrating peptides for nucleic acid and drug delivery in cancer. Biochim. Biophys. Acta.

[8] Koren E., Torchilin V.P. (2012). Cell-penetrating peptides: breaking through to the other side. Trends Mol. Med..

[9] Lehto T., Kurrikoff K., Langel U. (2012). Cell-penetrating peptides for the delivery of nucleic acids. Expert Opin. Drug Deliv..

[10] Bruno J.G., Carrillo M.P., Crowell R. (2009). Preliminary development of DNA aptamer-Fc conjugate opsonins. J. Biomed. Mat. Res. A.

[11] Bruno J.G., Carrillo M.P., Phillips T. (2008). *In vitro* antibacterial effects of anti-lipopolysaccharide DNA aptamer-C1qrs complexes. Folia Microbiol..

[12] Bruno J.G. (2010). Aptamer-biotin-streptavidin-C1q complexes can trigger the classical complement pathway to kill cancer cells. In Vitro Cell. Dev. Biol..

[13] Bruno J.G., Miner J.C. (2013). Therapeutic nucleic acid-3'—Conjugates. U.S. Patent.

[14] Hakulinen J., Meri S. (1998). Complement-mediated killing of microtumors *in vitro*. Am. J. Path..

[15] Stecker J.R., Savage A., Bruno J.G., Garcia D.M., Koke J.R. (2012). Dynamics and visualization of MCF7 adenocarcinoma cell death by aptamer-C1q-mediated membrane attack. Nucleic Acid Ther..

[16] Chu T.C., Marks J.W., Lavery L.A., Faulkner S., Rosenblum M.G., Ellington A.D., Levy M. (2006). Aptamer:toxin conjugates that specifically target prostate tumor cells. Cancer Res..

[17] Bagalkot V., Farokhzad O.C., Langer R., Jon S. (2006). An aptamer-doxorubicin physical conjugate as a novel targeted drug-delivery platform. Angew. Chem. Int. Ed. Eng..

[18] Huang Y.F., Shangguan D., Liu H., Phillips J.A., Zhang X., Chen Y., Tan W. (2009). Molecular assembly of an aptamer-drug conjugate for targeted drug delivery to tumor cells. Chembiochem..

[19] Tan W., Wang H., Chen Y., Zhang X., Zhu H., Yang C., Yang R., Liu C. (2011). Molecular aptamers for drug delivery. Trends Biotechnol..

[20] Borbas K.E., Ferreira C.S.M., Perkins A., Bruce J.I., Missailidis S. (2007). Design and synthesis of mono- and multimeric targeted radiopharmaceuticals based on novel cyclen ligands coupled to anti-MUC1 aptamers for the diagnostic imaging and targeted radiotherapy of cancer. Bioconj. Chem..

[21] Da Pieve C.D., Perkins A.C., Missailidis S. (2009). Anti-MUC1 aptamers: radiolabelling with (99m) Tc and biodistribution in MCF-7 tumour-bearing mice. Nucl. Med. Biol..

[22] Bakalova R., Ohba H., Zhelev Z., Ishikawa M., Baba Y. (2004). Quantum dots as photosensitizers?. Nat. Biotechnol..

[23] Dwarakanath S., Bruno J.G., Athmaram T.N., Bali G., Vattem D., Rao P. (2007). Antibody-quantum dot conjugates exhibit enhanced antibacterial effect * vs.* unconjugated quantum dots. Folia Microbiol..

[24] Ferreira C.S., Cheung M.C., Missailidis S., Bisland S., Gariépy J. (2009). Phototoxic aptamers selectively enter and kill epithelial cancer cells. Nucleic Acids Res..

[25] Jin T., Sun D., Su J.Y., Zhang H., Sue H.J. (2009). Antimicrobial efficacy of zinc oxide quantum dots against *Listeria monocytogenes*, *Salmonella enteritidis*, and *Escherichia coli* O157:H7. J. Food Sci..

[26] Samia A.C., Dayal S., Burda C. (2006). Quantum dot-based energy transfer: perspectives and potential for applications in photodynamic therapy. Photochem. Photobiol..

[27] Shi L., Hernandez B., Selke M. (2006). Singlet oxygen generation from water-soluble quantum dot-organic dye nanocomposites. J. Am. Chem. Soc..

[28] Yang X., Huang J., Wang K., Li W., Cui L., Li X. (2011). Angiogenin-mediated photosensitizer-aptamer conjugate for photodynamic therapy. Chem. Med. Chem..

[29] Yang X., Liu X., Liu Z., Pu F., Ren J., Qu X. (2012). Near-infrared light-triggered, targeted drug delivery to cancer cells by aptamer-gated nanovehicles. Adv. Mater..

[30] Beqa L., Fan Z., Singh A.K., Senapati D., Ray P.C. (2011). Gold nano-popcorn attached SWCNT hybrid nanomaterial for targeted diagnosis and photothermal therapy of human breast cancer cells. ACS Appl. Mater. Interfaces.

[31] Farokhzad O.C., Cheng J., Teply B.A., Sherifi I., Jon S., Kantoff P.W., Richie J.P., Langer R. (2006). Targeted nanoparticle-aptamer bioconjugates for cancer chemotherapy *in vivo*. Proc. Natl. Acad. Sci. USA.

[32] Huang Y.F., Chang H.T., Tan W. (2008). Cancer cell targeting using multiple aptamers conjugated on nanorods. Anal. Chem..

[33] Huang Y.F., Sefah K., Bamrungsap S., Chang H.T., Tan W. (2008). Selective photothermal therapy for mixed cancer cells using aptamer-conjugated nanorods. Langmuir.

[34] Li L.L., Yin Q., Cheng J., Lu Y. (2012). Polyvalent mesoporous silica nanoparticle-aptamer bioconjugates target breast cancer cells. Adv. Health Mater..

[35] Liu S., Wei L., Hao L., Fang N., Wook Chang M., Xu R., Yang Y., Chen Y. (2009). Sharper and faster “nano darts” kill more bacteria: A study of antibacterial activity of individually dispersed pristine single-walled carbon nanotube. ACS Nano.

[36] Wang J., Zhu G., You M., Song E., Shukoor M.I., Zhang K., Altman M.B., Chen Y., Zhu Z., Huang C.Z. (2012). Assembly of aptamer switch probes and photosensitizer on gold nanorods for targeted photothermal and photodynamic cancer therapy. ACS Nano.

[37] Yang L., Zhang X., Ye M., Jiang J., Yang R., Fu T., Chen Y., Wang K., Liu C., Tan W. (2011). Aptamer-conjugated nanomaterials and their applications. Adv. Drug Deliv. Rev..

[38] Berezhnoy A., Brenneman R., Bajgelman M., Seales D., Gilboa E. (2012). Thermal stability of siRNA modulates aptamer-conjugated siRNA inhibition. Mol. Ther. Nucl. Acids.

[39] Dassie J.P., Liu X.Y., Thomas G.S., Whitaker R.M., Thiel K.W., Stockdale K.R., Meyerholz D.K., McCaffrey A.P., McNamara J.O., Giangrande P.H. (2009). Systemic administration of optimized aptamer-siRNA chimeras promotes regression of PSMA-expressing tumors. Nat. Biotechnol..

[40] Neff C.P., Zhou J., Remling L., Kuruvilla J., Zhang J., Li H., Smith D.D., Swiderski P., Rossi J.J., Akkina R. (2011). An aptamer-siRNA chimera suppresses HIV-1 viral loads and protects from helper CD4 (+) T cell decline in humanized mice. Sci. Transl. Med..

[41] Zhou J., Bobbin M.L., Burnett J.C., Rossi J.J. (2012). Current progress of RNA aptamer-based therapeutics. Front. Genet..

[42] Zhou J., Rossi J.J. (2010). Aptamer-targeted cell-specific RNA interference. Silence.

[43] Bruno J.G., Crowell R. (2008). Selective glutaraldehyde-mediated coupling of proteins to the 3' adenine terminus of Polymerase Chain Reaction products. J. Biomolec. Techn..

[44] Bruno J.G., Stecker J.R., Carrillo M.P., Phillips T., Savage A., Garcia D.M., Koke J.R., Bruno J.G. (2013). Chapter 4: Novel aptamer-based therapeutic strategies. Biomedical Applications of Aptamers.

[45] Aarons L., Grennan D.M., Siddiqui M. (1983). The binding of ibuprofen to plasma proteins. Eur. J. Clin. Pharmacol..

[46] Cheruvallath V.K., Riley C.M., Narayanan S.R., Lindenbaum S., Perrin J.H. (1997). A quantitative circular dichroic investigation of the binding of the enantiomers of ibuprofen and naproxen to human serum albumin. J. Pharm. Biomed. Anal..

[47] Manoharan M., Rajeev K.G., Kesavan V. (2009). Single-stranded and double-stranded oligonucleotides comprising a 2-arylpropyl moiety. U.S. Patent Application.

[48] Stasiak P., Sznitowska M., Ehrhardt C., Luczyk-Juzwa M., Grieb P. (2010). *In vivo* assessment of parenteral formulations of oligo (3-hydroxybutyric acid) conjugates with the model compound ibuprofen. AAPS Pharm. Sci. Tech..

[49] Zion T.C., Lancaster T.M. (2011). Polynucleotide aptamers-based cross-linked materials and uses thereof. U.S. Patent Application.

[50] Zsila F., Bikadi Z., Malik D., Hari P., Pechan I., Berces A., Hazai E. (2011). Evaluation of drug-human serum albumin binding interactions with support vector machine aided online automated docking. Bioinformatics.

[51] Wright S.E. (2012). Immunotherapy of breast cancer. Expert Opin. Biol. Ther..

[52] Wu A.M., Senter P.D. (2005). Arming antibodies: prospects and challenges for immunoconjugates. Nat. Biotechnol..

[53] Bruno J.G., Carrillo M.P., Richarte A.M., Phillips T., Andrews C., Lee J.S. (2012). Development, screening, and analysis of a small DNA aptamer library potentially useful for diagnosis and passive immunity of arboviruses. BMC Res. Notes.

[54] Chen F., Zhou J., Huang Y.H., Huang F.Y., Liu Q., Fang Z., Yang S., Xiong M., Lin Y.Y., Tan G.H. (2013). Function of ssDNA aptamer and aptamer pool against *Mycobacterium tuberculosis* in a mouse model. Mol. Med. Rep..

[55] Cheng C., Dong J., Yao L., Chen A., Jia R., Huan L., Guo J., Shu Y., Zhang Z. (2008). Potent inhibition of human influenza H5N1 virus by oligonucleotides derived by SELEX. Biochem. Biophys. Res. Commun..

[56] Mullis K.B. Chemically programmable immunity. U.S. Patent.

[57] Kiel J.L., Kiel A.L., Bruno J.G. (2013). Chapter 1: Fundamental performance differences between antibodies and aptamers. Biomedical Applications of Aptamers.

[58] Fan S., Wu F., Martiniuk F., Hale M.L., Ellington A.D., Tchou-Wong K.M. (2008). Protective effects of anti-ricin A-chain RNA aptamer against ricin toxicity. World J. Gastroenterol..

[59] Lauridsen L.H., Veedu R.N. (2012). Nucleic acid aptamers against biotoxins: A new paradigm toward the treatment and diagnostic approach. Nucleic Acid Ther..

[60] Dobler R.K., Maki W.C. Mars health care delivery systems: Aptamers provide critical technology. 12th NASA Symposium of VLSA Design.

[61] Healy J.M., Lewis S.D., Kurz M., Boomer R.M., Thompson K.M., Wilson C., McCauley T.G. (2004). Pharmacokinetics and biodistribution of novel aptamer compositions. Pharm. Res..

[62] King D.J., Ventura D.A., Brasier A.R., Gorenstein D.G. (1998). Novel combinatorial selection of phosphorothioate oligonucleotide aptamers. Biochemistry.

[63] Cload S.T., McCauley T.G., Keefe A.D., Healy J.M., Wilson C., Klussmann S. (2006). Chapter 17: Properties of therapeutic aptamers. The Aptamer. Handbook.

[64] Eulberg D., Klussmann S. (2003). Spiegelmers: Biostable aptamers. Chembiochem.

[65] Eulberg D., Jarosch F., Vanhoff S., Klussmann S., Klussmann S. (2006). Chap 18: Spiegelmers for therapeutic applications—Use of chiral principles in evolutionary selection techniques. The Aptamer. Handbook.

[66] Boomer R.M., Lewis S.D., Healy J.M., Kurz M., Wilson C., McCauley T.G. (2005). Conjugation to polyethylene glycol polymer promotes aptamer biodistribution to healthy and inflamed tissues. Oligonucleotides.

[67] Da Pieve C., Blackshaw E., Missailidis S., Perkins A.C. (2012). PEGylation and biodistribution of an anti-MUC1 aptamer in MCF-7 tumor-bearing mice. Bioconjug. Chem..

[68] Jäschke A., Fürste J.P., Nordhoff E., Hillenkamp F., Cech D., Erdmann V.A. (1994). Synthesis and properties of oligodeoxyribonucleotide-polyethylene glycol conjugates. Nucleic Acids Res..

[69] Dougan H., Lyster D.M., Vo C.V., Stafford A., Weitz J.I., Hobbs J.B. (2000). Extending the lifetime of anticoagulant oligodeoxynucleotide aptamers in blood. Nucl. Med. Biol..

[70] Farkas I., Baranyi L., Ishikawa Y., Okada N., Bohata C., Budai D., Fukuda A., Imai M., Okada H. (2002). CD59 blocks not only the insertion of C9 into MAC but inhibits ion channel formation by homologous C5b-8 as well as C5b-9. J. Physiol..

[71] Dominguez M., Moreno I., Aispurua C., Torano A. (2003). Early mechanisms of *Leishmania.* infection in human blood. Microbes Infect..

[72] Carter J.D., LaBean T.H. (2011). Coupling strategies for the synthesis of peptide-oligonucleotide conjugates for patterned synthetic biomineralization. J. Nucleic Acids..

[73] Hasegawa H., Taira K., Sode K., Ikebukuro K. (2008). Improvement of aptamer affinity by dimerization. Sensors.

[74] Mallikaratchy P.R., Ruggiero A., Gardner J.R., Kuryavyi V., Maguire W.F., Heaney M.L., McDevitt M.R., Patel D.J., Scheinberg D.A. (2011). A multivalent DNA aptamer specific for the B-cell receptor on human lymphoma and leukemia. Nucleic Acids Res..

[75] Tian L., Heyduk T. (2009). Bivalent ligands with long nanometer-scale flexible linkers. Biochemistry.

[76] Yang L., Meng L., Zhang X., Chen Y., Zhu G., Liu H., Xiong X., Sefah K., Tan W. (2011). Engineering polymeric aptamers for selective cytotoxicity. J. Am. Chem. Soc..

[77] Murray E., Gregg D.A., Norton M.L., Swick J.T., Towler W.I. (2012). Method for a continuous rapid thermal cycle system. U.S. Patent.

[78] Wahajuddin S.A., Arora S. (2012). Superparamagnetic iron oxide nanoparticles: Magnetic nanoplatforms as drug carriers. Int. J. Nanomed..

[79] Nair B.G., Nagaoka Y., Morimoto H., Yoshida Y., Maekawa T., Kumar D.S. (2010). Aptamer conjugated magnetic nanoparticles as nanosurgeons. Nanotechnology.

[80] Silva A.C., Oliveira T.R., Mamani J.B., Malheiros S.M., Malavolta L., Pavon L.F., Sibov T.T., Amaro E., Tannús A., Vidoto E.L. (2011). Application of hyperthermia induced by superparamagnetic iron oxide nanoparticles in glioma treatment. Int. J. Nanomed..

[81] Hilger I., Kaiser W.A. (2012). Iron oxide-based nanostructures for MRI and magnetic hyperthermia. Nanomedicine.

[82] Kobayashi T. (2011). Cancer hyperthermia using magnetic nanoparticles. Biotechnol. J..

[83] Cavaliere R., Ciocatto E.C., Giovanella B.C., Heidelberger C., Johnson R.O., Margottini M., Mondovi B., Moricca G., Rossi-Fanelli A. (1967). Selective heat sensitivity of cancer cells. Biochemical and clinical studies. Cancer.

[84] Christophi C., Winkworth A., Muralihdaran V., Evans P. (1998). The treatment of malignancy by hyperthermia. Surg. Oncol..

[85] Sellins K.S., Cohen J.J. (1991). Hyperthermia induces apoptosis in thymocytes. Radiat. Res..

[86] Glazer E.S., Curley S.A. (2011). Non-invasive radiofrequency ablation of malignancies mediated by quantum dots, gold nanoparticles and carbon nanotubes. Ther. Deliv..

[87] Glazer E.S., Curley S.A. (2010). Radiofrequency field-induced thermal cytotoxicity in cancer cells treated with fluorescent nanoparticles. Cancer.

[88] Glazer E.S., Zhu C., Massey K.L., Thompson C.S., Kaluarachchi W.D., Hamir A.N., Curley S.A. (2010). Noninvasive radiofrequency field destruction of pancreatic adenocarcinoma xenografts treated with targeted gold nanoparticles. Clin. Cancer Res..

[89] Wang Q., Fang T., Liu P., Min X., Li X. (2011). Study of the bioeffects of CdTe quantum dots on *Escherichia coli* cells. J. Coll. Interface Sci..

[90] Liu W., Zhang S., Wang L., Qu C., Zhang C., Hong L., Yuan L., Huang Z., Wang Z., Liu S., Jiang G. (2011). CdSe quantum dot (QD)-induced morphological and functional impairments to liver in mice. PLoS One.

[91] Li K.G., Chen J.T., Bai S.S., Wen X., Song S.Y., Yu Q., Li J., Wang Y.Q. (2009). Intracellular oxidative stress and cadmium ions release induce cytotoxicity of unmodified cadmium sulfide quantum dots. Toxicol. In Vitro..

[92] Tsay J.M., Trzoss M., Shi L., Kong X., Selke M., Jung M.E., Weiss S. (2007). Singlet oxygen production by peptide-coated quantum dot-photosensitizer conjugates. J. Am. Chem. Soc..

[93] Yaghini E., Seifalian A.M., MacRobert A.J. (2009). Quantum dots and their potential biomedical applications in photosensitization for photodynamic therapy. Nanomedicine.

[94] Savla R., Taratula O., Garbuzenko O., Minko T. (2011). Tumor targeted quantum dot-mucin 1 aptamer-doxorubicin conjugate for imaging and treatment of cancer. J. Control. Release.

[95] Taghdisi S.M., Lavaee P., Ramezani M., Abnous K. (2011). Reversible targeting and controlled release delivery of daunorubicin to cancer cells by aptamer-wrapped carbon nanotubes. Eur. J. Pharm. Biopharm..

[96] Tan W., Wang H., Chen Y., Zhang X., Zhu H., Yang C., Yang R., Liu C. (2011). Molecular aptamers for drug delivery. Trends Biotechnol..

[97] Lu W., Singh A.K., Khan S.A., Senapati D., Yu H., Ray P.C. (2010). Gold nano-popcorn-based targeted diagnosis, nanotherapy treatment, and *in situ* monitoring of photothermal therapy response of prostate cancer cells using surface-enhanced Raman spectroscopy. J. Am. Chem. Soc..

[98] Jarmila V., Vavríková E. (2011). Chitosan derivatives with antimicrobial, antitumour and antioxidant activities-a review. Curr. Pharm. Des..

[99] Patel M.P., Patel R.R., Patel J.K. (2010). Chitosan mediated targeted drug delivery system: A review. J. Pharm. Pharmaceut. Sci..

[100] Bowman K., Leong K.W. (2006). Chitosan nanoparticles for oral drug and gene delivery. Int. J. Nanomedicine.

[101] Hassan E.E., Gallo J.M. (1993). Targeting anticancer drugs to the brain. I: Enhanced brain delivery of oxantrazole following administration in magnetic cationic microspheres. J. Drug Target..

[102] Tallury P., Kar S., Bamrungsap S., Huang Y.F., Tan W., Santra S. (2009). Ultra-small water dispersible fluorescent chitosan nanoparticles: Synthesis, characterization and specific targeting. Chem. Commun..

[103] Mann A.P., Bhavane R.C., Somasunderam A., Montalvo-Ortiz B., Ghaghada K.B., Volk D., Nieves-Alicea R., Suh K.S., Ferrari M., Annapragada A. (2011). Thioaptamer conjugated liposomes for tumor vasculature targeting. Oncotarget.

[104] Kolhe P., Khandare J., Pillai O., Kannan S., Lieh-Lai M., Kannan R. (2004). Hyperbranched polymer-drug conjugates with high drug payload for enhanced cellular delivery. Pharm. Res..

[105] Kolhe P., Khandare J., Pillai O., Kannan S., Lieh-Lai M., Kannan R.M. (2006). Preparation, cellular transport, and activity of polyamidoamine-based dendritic nanodevices with a high drug payload. Biomaterials.

[106] Kim Y., Cao Z., Tan W. (2008). Molecular assembly for high-performance bivalent nucleic acid inhibitor. Proc. Natl. Acad. Sci. USA.

[107] McNamara J.O., Kolonias D., Pastor F., Mittler R.S., Chen L., Giangrande P.H., Sullenger B., Gilboa E. (2008). Multivalent 4-1BB binding aptamers costimulate CD8+ T cells and inhibit tumor growth in mice. J. Clin. Invest..

[108] Wachowius F., Höbartner C. (2011). Probing essential nucleobase functional groups in aptamers and deoxyribozymes by nucleotide analogue interference mapping of DNA. J. Am. Chem. Soc..

[109] Hollenstein M. (2011). Expanding the catalytic repertoire of DNAzymes by modified nucleosides. ChimiaInt. J. Chem..

[110] Sanghvi Y.S. (2011). A status update of modified oligonucleotides for chemotherapeutics applications. Curr. Protoc. Nucleic Acid Chem..

[111] Sundaram P., Kurniawan H., Byrne M.E., Wower J. (2013). Therapeutic RNA aptamers in clinical trials. Eur. J. Pharm. Sci..

